# Dose‐response effects of light at night on the reproductive physiology of great tits (*Parus major*): Integrating morphological analyses with candidate gene expression

**DOI:** 10.1002/jez.2214

**Published:** 2018-07-29

**Authors:** Davide M. Dominoni, Maaike de Jong, Michelle Bellingham, Peter O'Shaughnessy, Kees van Oers, Jane Robinson, Bethany Smith, Marcel E. Visser, Barbara Helm

**Affiliations:** ^1^ Institute of Biodiversity, Animal Health and Comparative Medicine University of Glasgow Glasgow UK; ^2^ Department of Animal Ecology Netherlands Institute of Ecology (NIOO‐KNAW) Wageningen The Netherlands; ^3^ GELIFES, Groningen Institute for Evolutionary Life Sciences University of Groningen Groningen The Netherlands

**Keywords:** ALAN, HPG axis, spermatogenesis, testis, timing of reproduction, urbanization

## Abstract

Artificial light at night (ALAN) is increasingly recognized as a potential threat to wildlife and ecosystem health. Among the ecological effects of ALAN, changes in reproductive timing are frequently reported, but the mechanisms underlying this relationship are still poorly understood. Here, we experimentally investigated these mechanisms by assessing dose‐dependent photoperiodic responses to ALAN in the great tit (*Parus major*). We individually exposed photosensitive male birds to one of three nocturnal light levels (0.5, 1.5, and 5 lux), or to a dark control. Subsequent histological and molecular analyses on their testes indicated a dose‐dependent reproductive response to ALAN. Specifically, different stages of gonadal growth were activated after exposure to different levels of light at night. mRNA transcript levels of genes linked to the development of germ cells (*stra8* and *spo11*) were increased under 0.5 lux compared to the dark control. The 0.5 and 1.5 lux groups showed slight increases in testis size and transcript levels associated with steroid synthesis (*lhr* and *hsd3b1*) and spermatogenesis (*fshr, wt1, sox9*, and *cldn11*), although spermatogenesis was not detected in histological analysis. In contrast, all birds under 5 lux had 10 to 30 times larger testes than birds in all other groups, with a parallel strong increase in mRNA transcript levels and clear signs of spermatogenesis. Across treatments, the volume of the testes was generally a good predictor of testicular transcript levels. Overall, our findings indicate that even small changes in nocturnal light intensity can increase, or decrease, effects on the reproductive physiology of wild organisms.

## 1 INTRODUCTION

Artificial light at night (ALAN) is one of the most evident anthropogenic modifications of the natural environment (Falchi et al., 2016; Kyba et al., 2017). As natural and rural lands are increasingly converted into urban areas globally, and in particular in developing countries, the proportion of the Earth exposed to ALAN is also increasing (Falchi et al., 2016; Gaston, Visser, & Hölker, 2015b; Kyba et al., 2017). This increase has been recently quantified as 6% per year worldwide (Falchi et al., 2016). The presence of light pollution alters natural regimes of light and darkness (Davies, Bennie, Inger, & Gaston, 2013), and this can have important consequences for human health and economy (Gaston, Gaston, Bennie, & Hopkins, 2014). The ecological consequences of ALAN were first recognized in the early 20th century (Rowan, 1937), but have only recently become an important focus of scientific research. ALAN is being increasingly associated with changes in behavior, physiology, and life histories of wild organisms, from plants to invertebrates, fishes, amphibians, reptiles, birds, and mammals (Bennie, Davies, Cruse, & Gaston, 2016; Bruening, Hölker, & Wolter, 2011; Da Silva, Valcu, & Kempenaers, 2015; Davies, Bennie, & Gaston, 2012; Jones, Durrant, Michaelides, & Green, 2015; Knop et al., 2017; Perry, Buchanan, & Fisher, 2008; Spoelstra et al., 2017).

The effects of ALAN on seasonal cycles, for instance reproduction, have been a key focus of light pollution research (Bruening, Hölker, Franke, Kleiner, & Kloas, 2016; Dominoni, 2015; Gaston, Davies, Nedelec, & Holt, 2017; Robert et al., 2015). For example, clear evidence for a direct effect of ALAN on reproduction comes from experimental studies on birds in controlled environments. European blackbirds (*Turdus merula*) exposed to 0.3 lux of ALAN began to grow their gonads 3 weeks earlier than conspecifics exposed to dark nights (Dominoni, Quetting, & Partecke, 2013a). Birds have also been the focus of experimental studies in the field, although these have not measured gonadal growth. For instance, great tits (*Parus major*) living in areas polluted with white LEDs laid their eggs 5 days earlier than in dark areas, although only in cold springs (de Jong et al., 2015). In addition, a correlational study has shown that female blue tits (*Cyanistes careuleus*) breeding in proximity of street lights laid their first egg 1.5 days earlier than females breeding in dark areas (Kempenaers, Borgström, Loës, Schlicht, & Valcu, 2010). Other field studies have focused on the phenology of dawn song and morning activity. These studies have shown that ALAN is associated with an advancement of dawn song earlier in the morning (with the exception of Da Silva et al., 2017), and also earlier in the season, an effect that is suggestive of an earlier propensity to breed (Da Silva et al., 2015; Dominoni & Partecke, 2015; Kempenaers et al., 2010; Miller, 2006; Nordt & Klenke, 2013). In contrast to advanced reproductive behaviors in birds, other taxa have been shown to delay reproduction in response to ALAN, presumably as a consequence of short‐day breeding. Examples include delayed average birth time of tammar wallabies (*Macropus eugenii*) (Robert, Lesku, Partecke, & Chambers, 2015) or completely inhibited reproductive physiology of adult perch (*Perca fluviatilis*) (Bruening et al., 2016). Similarly, in insects, ALAN has been shown to disrupt mating behavior and delay the time to pupation of moths (van Geffen, van Grunsven, van Ruijven, Berendse, & Veenendaal, 2014, 2015).

Effects of ALAN on reproduction are intuitive given that most organisms use photoperiod to time the development of their reproductive system in anticipation of the expected annual peak in resource availability (Bradshaw & Holzapfel, 2010; Helm et al., 2013). Use of reliable timing cues is particularly important for animals that substantially regrow their reproductive organs on an annual basis, such as birds, where the process of gonadal development requires several weeks to be completed. In temperate areas, for long‐day breeders, the increasing day length of late winter/early spring is the best proximate cue to start gonadal development in advance of breeding (Dawson, King, Bentley, & Ball, 2001). This is because day length, unlike temperature and food availability, shows little inter‐annual variation. Experimental exposure to long days causes birds to enter into reproductive state relatively quickly (Follett & Sharp, 1969; Follett, Mattocks, & Farner, 1974; Rowan, 1938; te Marvelde, Schaper, & Visser, 2012). However, other environmental factors, such as temperature and food availability, fine‐tune the final breeding decisions, in particular the timing of egg‐laying (Ball, 2012; Schoech & Hahn, 2007; Schaper et al., 2012b). Therefore, variation in timing of reproductive physiology does not necessarily predict variation in egg‐laying dates, but defines its scope (Schaper et al., 2012a; te Marvelde et al., 2012).

**Figure 1:**
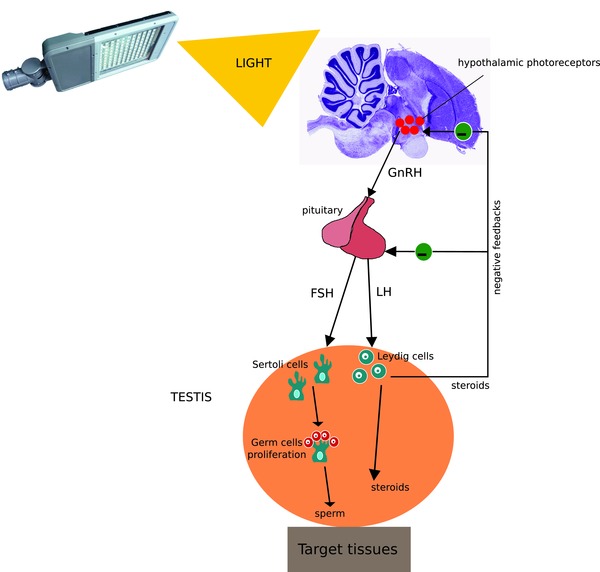
Simplified scheme illustrating the structure and function of the avian HPG axis. Light stimulates deep‐brain photoreceptors, which promote the release of GnRH from the hypothalamus to the anterior pituitary. This releases FSH and LH into the blood circulation, which then arrive at the testes binding to their receptors located on Sertoli and Leydig cells, respectively. Sertoli cells stimulate the rapid and massive proliferation of germ cells, which then lead to sperm production, while Leydig cells stimulate the production of steroids. Steroid hormones, such as testosterone, negatively feedback on the hypothalamus and pituitary to stop further release of GnRH, FSH and LH [Color figure can be viewed at http://wileyonlinelibrary.com]

In birds, reproductive activation is mediated by the hypothalamus–pituitary–gonadal (HPG) axis via photo‐stimulation of deep‐brain photoreceptors. Increasing day lengths stimulate the secretion of gonadotropin‐release hormone (GnRH) in the hypothalamus (Nakane et al., 2010). GnRH then activates the pituitary gland that releases follicle‐stimulating hormone (FSH) and luteinizing hormone (LH) into the blood stream (Dawson, 2002; Sharp, 2005; Figure 1). LH and FSH bind to their specific receptors (*lhr* and *fshr*). In the males’ testes, they activate Leydig and Sertoli cells, respectively (Brown, Baylé, Scanes, & Follett, 1975). LH stimulates Leydig cells to produce androgens, mediated by enzymes, such as *cyp11a1*, *cyp17a1*, as well as several dehydrogenases (for instance *hsd3b1*, required for progesterone production, and *hsd17b3*, involved in the synthesis of testosterone) (Brown et al., 1975; Purcell and Wilson, 1975). Sertoli cells are somatic cells that are essential for the development of the testis and spermatogenesis (Figure 1; Thurston & Korn, 2000). They are located in the seminiferous tubules of the testis and promote spermatogenesis through formation of a blood‐testis barrier and through direct interactions with the developing germ cells. Sertoli cell activity depends on FSH stimulation and on androgens secreted by the Leydig cells (da Silva et al., 1996; Thurston & Korn, 2000). In the absence of hormone stimulation of the Sertoli cells, germ cell development does not progress beyond early meiosis (O'Shaughnessy, 2014).

During gonadal development in birds, transcript levels of glucocorticoid (*nr3c1*, also referred to as *gr*) and mineralocorticoid receptors (*nr3c2*, also referred to as *mr*) also increase (Fudickar et al., 2017; Kirby, Geraghty, Ubuka, Bentley, & Kaufer, 2009; Lattin et al., 2012; McGuire, Koh, & Bentley, 2013). This provides a mechanistic basis for the widespread links between adrenal steroids and timing of reproduction and reproductive investment reported in several bird species (Crespi, Williams, Jessop, & Delehanty, 2013; Deviche et al., 2010; Goutte et al., 2010a; Lattin, Breuner, & Michael Romero, 2016; McGuire et al., 2013; Schoech, Rensel, Bridge, Boughton, & Wilcoxen, 2009). Indeed, during stressful periods, sex steroid production and spermatogenesis can be suppressed via several routes, one of which is by increased glucocorticoid levels (Blas, 2015; Hazra et al., 2014; McGuire et al., 2013; Witorsch, 2016). Since ALAN has been linked to higher baseline corticosterone levels in birds (Ouyang et al., 2015), it could also influence reproductive activation, although it would be expected to slow, not accelerate, testicular development. Alternatively, upregulation of gonadal receptors for adrenal steroids could be a mechanism to enhance growth of the reproductive system, which is an energetically costly process requiring metabolic activation by adrenal steroids (Wingfield & Farner, 1993).

Despite the increasing interest in the effects of light pollution on reproduction of wild animals, we have still little understanding of the sensitivity of day length detection and of the ensuing sequence of reproductive activation events to ALAN. The aim of our study was thus to investigate the sensitivity of different stages of gonadal development to a realistic range of light intensities, derived from recordings from free‐living birds (Dominoni et al., 2013a). We tested for the existence of dose‐dependent, or alternatively, threshold responses, of the reproductive system to increasing levels of ALAN. As study subject we chose a songbird, the great tit, which was previously reported to show behavioral responses to similar levels of ALAN (de Jong et al., 2016a). We used a between‐animal design in which we exposed captive male great tits to three different nocturnal light treatments (0.5, 1.5, and 5 lux) or to dark nights in late winter. After 3 weeks of exposure, birds were sacrificed and their testes were collected. We recorded testicular sizes for comparison with laparotomy data measured in field and captivity studies of living birds (e.g., (Partecke, Van't Hof, & Gwinner, 2005)). These measures were complemented by morphological, histological, and molecular analyses. We assessed mRNA transcript levels in the testis for 10 candidate genes implicated in morphological development of the reproductive system, in synthesis of steroids and in promotion of spermatogenesis (Table 1). More specifically, these genes are involved in germ cell development (*stra8* and *spo11*), Sertoli cell activation, and spermatogenesis (*fshr, wt1, sox9, cldn11*), Leydig cell activation and steroid synthesis (*lhr* and *hsd3b1*), and adrenal steroid function (*gr* and *mr*) (Table 1, Figure 1).

**Table 1:** List of gene transcripts measured and function of the relevant protein

General function	Gene acronym	Specific function	References
*Germ cells development*			
	stra8	Regulation of meiotic initiation	(Krentz et al., 2011; Zhang et al., 2015)
	spo11	Involved in meiotic recombination	(Guioli et al., 2014; Oréal, Mazaud, Picard, Magre, & Carré‐Eusèbe, 2002)
*Sertoli cells activity (spermatogenesis)*			
	fshr	Gonadotropin receptor, stimulates spermatogenesis	(Akazome, Abe, & Mori, 2002; Yamamura et al., 2000)
	wt1	Marker of Sertoli cells activity	(Kent, Coriat, Sharpe, Hastie, & van Heyningen, 1995; Oréal et al., 2002)
	sox9	Required for Sertoli cell differentiation and marker of adult Sertoli cell	(da Silva et al., 1996; Lee, Kim, Kim, Song, & Lim, 2007)
	cldn11	Involved in the formation of tight junctions (blood testis barrier)	(Gunzel & Yu, 2013)
*Leydig cells activity (steroid synthesis)*			
	lhr	Leydig cell gonadotropin receptor, stimulates steroid synthesis	(Akazome et al., 2002; Yamamura et al., 2000)
	hsd3b1	Enzyme required for androgen synthesis	(Lee et al., 2007; London & Clayton, 2010)
*Adrenal steroid receptors*			
	nr3c1 (gr)	Glucocorticoid receptor	(Landys, Piersma, Ramenofsky, & Wingfield, 2004; Liebl & Martin, 2013)
Stress and metabolism	nr3c2 (mr)	Mineralocorticoid receptor	(Landys et al., 2004; Liebl & Martin, 2013)

Overall, we hypothesized that:

1. Increasing light intensity at night would lead to larger testicular volume and advanced morphological differentiation.
2. Changes in size and morphology would be paralleled by changes in transcript levels of functionally related genes. Higher light levels should progressively activate early stages of gonadal development, such as germ cells development (*stra8* and *spo11)*, activation of Sertoli (*fshr, wt1, sox9*, and *cldn11*) and Leydig cells (*lhR* and *hsd3b1*), and complete spermatogenesis. Whether or not a set of functionally related genes would show increased mRNA levels would depend on light intensity.
3. Since reproductive activation has been linked to an increased expression of adrenal steroid receptors, we also expect increased ALAN levels to be associated with increased adrenal steroid receptors. Alternatively, reproductive activation could be this association could be counteracted by elevated circulating CORT levels, which have been associated to ALAN.
4. Levels of mRNA transcripts encoding cell‐specific proteins would be related to testis size at the individual level.

## 2 MATERIALS AND METHODS

### 2.1 Animals and experimental setup

We conducted the experiment between February 1 and February 23, 2014. We used 34 adult male great tits that had been used in a previous experiment aimed at assessing the impact of different levels of light intensity at night on daily activity and physiology (de Jong et al., 2016a). All birds had been hand‐raised and housed at the Netherlands Institute of Ecology, Wageningen, The Netherlands, in individual cages (90 × 50 × 40 cm). All birds were between 1 and 4 years of age (hatched in 2012 or before), but mean age did not differ between treatment (*P* = 0.576). Temperature was maintained between 10 and 14°C, and did not vary structurally between day‐ and night‐time. Birds had access to food and water ad libitum.

During the ALAN experiment, birds were kept under fixed natural day‐length of 10 hr light and 14 hr darkness. Each cage had two separate light sources for day‐ and night‐time illumination. We used dividers between the cages, so that birds could only hear but not see each other, and light from one cage did not influence the light environment in adjacent cages because we had placed a wooden plate in the front of the cage. During the daytime, all birds were exposed to full spectrum daylight by high frequency fluorescent lights emitting ±1000 lux at perch level (Activa 172, Philips, Eindhoven, the Netherlands). During the night‐time, birds were assigned to different treatment groups that varied in the level of light intensity used (warm white LED light; Philips, Eindhoven, The Netherlands). The spectral composition of this light is shown in Supporting Information Figure S1 as part of the description of the preceding experiment mentioned in the Introduction (de Jong et al., 2016a). In this earlier experiment, the birds were exposed to five levels of ALAN (0.05, 0.15, 0.5, 1.5, and 5 lux) for 1 month between December 10, 2013 and January 10, 2014 and otherwise kept under dark nights. The experimental setup we used here differed in which we used four and not five experimental levels of ALAN, of which one was a dark control. The birds in the dark control were derived from the two earlier treatment groups with the lowest light intensity (0.05 and 0.15 lux, respectively), while birds in all other treatments were kept in the same treatment that they were exposed to in the previous experiment. Thus, from the start of our present experiment on February 1, 2014, the birds were exposed for the entire night to either one out of three nocturnal light intensities measured at perch level in the cages: 0.5 lux (*n* = 7), 1.5 lux (*n* = 7), or 5 lux (*n* = 7), or to dark control conditions (*n* = 13). For details on the spectral composition of lights, see the supplementary information of de Jong et al. (2016a).

The four treatment groups were assigned to one of seven blocks of cages arranged within two experimental rooms. Each block contained all treatment groups, distributed using a Latin Squares design. The birds were kept under these conditions for 3 weeks, and were then killed to collect tissues for morphological and molecular analyses. We sacrificed birds under isoflurane anesthesia (Forene, Abbott, Hoofddorp, the Netherlands) at midday (±2 hr) on February 22, 2014 or midnight (±2 hr) on both February 22 and 23, 2014. We used two sampling times to assess day–night differences in transcript levels for a separate study. Organs were extracted, snap‐frozen on dry ice, and stored at −80^o^C within 10 min of capture. Testes samples were then shipped to Glasgow, UK, in dry ice and stored at −80^o^C until further use. All experimental procedures were carried out under license NIOO 13.11 of the Animal Experimentation Committee (DEC) of the Royal Netherlands Academy of Arts and Sciences.

### 2.2 Morphological and histological analyses

On June 4, 2014, one frozen testis from each bird was placed into Neutral Buffered Formalin overnight to fix and preserve the tissue. Testes were weighed and their length and width measured before being placed into 70% ethanol. Testes were then embedded in wax, sectioned, and stained with hematoxylin and eosin for visualization of tissue structure. Tubule diameter and tubule area (where round tubular sections could be seen) were measured using ImageJ (NIH Image, https://imagej.net) and the development of spermatogenesis assessed.

We suspected that freezing might have caused the testes to change in size from the original, freshly measured state. We used measurements on fresh and frozen testes obtained from male great tits in another experiment to test this hypothesis (data are courtesy of Irene Verhagen). Testes length and width were highly correlated between fresh and frozen measurements (percentage change ± *SD*: width = 8.43 ± 9.84, length = 6.43 ± 11.13; *P* < 0.001; and *r* = 0.98 in both cases; *N* = 36; Supporting Information Figure S1).

### 2.3 Transcript analysis

The second testis was used for assessing mRNA transcript levels. Tissue was homogenized with a ribolyzer, and RNA extracted using 0.5 ml of Trizol (Thermofisher, UK) per sample. During the extraction, 5 ng of luciferase mRNA (Promega U.K., Southampton, UK) was added to each sample to serve as an external control (Rebourcet et al., 2014). Total RNA was reverse transcribed to generate cDNA using random hexamers and Moloney murine leukemia virus reverse transcriptase (Superscript III, Life Technologies) as described previously (O'Shaughnessy & Murphy, 1993).

Primers for the 10 candidate genes (Supporting Information Table S1) were designed using the great tit genome version 1.03 (Laine et al., 2016), and intron/exon boundaries were identified through BLAST against the zebra finch (*Taeniopygia guttata*) genome (Warren et al., 2010). Primers were designed using Primer ExpressTM 2.0.0 (Applied Biosystems) using parameters described previously (Czechowski, Bari, Stitt, Scheible, & Udvardi, 2004; O'Shaughnessy, Morris, & Baker, 2008). In order to avoid genomic DNA (gDNA) amplification, every primer pair was designed to span an intron of more than 1000 base pairs.

Real‐time PCR used the SYBR green method with a Stratagene MX3000 cycler 96‐well plates. Reactions contained 5 μl 2xSYBR mastermix (Agilent Technologies, Wokingham, UK), primers (100 nM), and template in a total volume of 10 μl. The thermal profile used for amplification was 95^o^C for 8 min followed by 40 cycles of 95^o^C for 25 s, 63^o^C for 25 s, and 72 ^o^C for 30 s. At the end of the amplification phase, a melting curve analysis was carried out on the products formed. We ran one transcript on each plate. Each sample was run in duplicate, and in each plate, we also included duplicate negative control wells (with RNA instead of cDNA). None of the primer pairs amplified gDNA and the efficiency of the qPCR reactions was always between 95% and 103%. Levels of transcripts encoding genes of interest were quantified relative to the luciferase external standard (luciferase) using the ΔCt method levels (Baker & O'Shaughnessy, 2001).

### 2.4 Statistical analyses

We ran all analyses in the statistical environment R (R Development Core Team, 2015). All models were linear mixed effects (LMMs) with block nested into room as random effect, to account for possible effects of location of the cage, and a Gaussian error structure. Treatment (continuous variable with four levels: dark (0 lux), 0.5, 1.5, and 5 lux), time (factor with two levels: day and night), the interaction of treatment and time, and age were always included as explanatory variables in the initial maximal models, unless specified otherwise. In the preliminary models, we also always included the second order polynomial effect of treatment to test for potential quadratic effect of increasing light intensities. Model selection was done by backward stepwise deletion of nonsignificant terms. When treatment or time were found to be significant, post hoc tests were done by comparing estimated marginal means and confidence intervals (CI) of the estimates for each level, using the function *emmeans* in the R package *emmeans (* https://cran.r-project.org/web/packages/emmeans/index.html *)*. Two levels were considered to be significantly different if the estimated marginal mean (Tukey's corrected) for one level was not included in the CI of another level. Assumptions for using linear models (normality and homogeneity of residuals) were met.

We first ran two separate LMMs to assess changes in morphology, with either testis volume or tubule diameter as response variables. In these models, we neither included time nor the interaction of time and treatment as explanatory variables, because we did not expect any change between day and night on the weekend that birds were sacrificed. We then ran four LMMs using grouped transcript levels as response variables. Genes were grouped based on their function as in Table 1, resulting in four groups: germ cells development (*stra8* and *spo11*), Sertoli cells activity (*fshr*, *wt1*, *sox9*, and *cldn11*), Leydig cells activity (*lhr* and *hsd3b1*) and adrenal steroid receptors (*gr* and *mr*). In these models individual ID was nested into block nested into room as random effect to correct for multiple transcript measurements of individuals in each model. Transcript levels were standardized within each group by calculating *Z*‐scores. To do so, we first calculated the mean and standard deviation of the entire vector of transcript levels within a functional group. Then, each value was recalculated by subtracting the mean and then divided by the standard deviation. Finally, we took a closer look at transcript levels of individual target genes by running 10 additional LMMs with the 10 target genes as response variables, log‐transformed.

To test for a potential relationship between testes size and gene transcript levels, we selected a single trait for testes size as response variable. Since testis volume was highly correlated to tubule diameter (*r* = 0.95 and *P* < 0.001), we decided to select testis volume because it is commonly taken on live birds through laparotomy, therefore enabling comparison with other studies. We then ran independent LMMs for each gene where transcript levels were the response variable, and testis volume, treatment, the quadratic effect of treatment, age, mass, and the interaction between testis volume and treatment were modeled as the explanatory variables.

## 3 RESULTS

### 3.1 Morphology and histology

**Figure 2:**
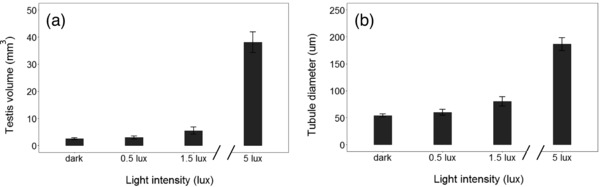
Light intensity at night (continuous variable) affects testis volume (a) and tubule diameter (b). Data are presented as mean ± *SEM*. Sample sizes were: dark = 13; 0.5 lux = 7; 1.5 lux = 7; 5 lux = 7

**Figure 3:**
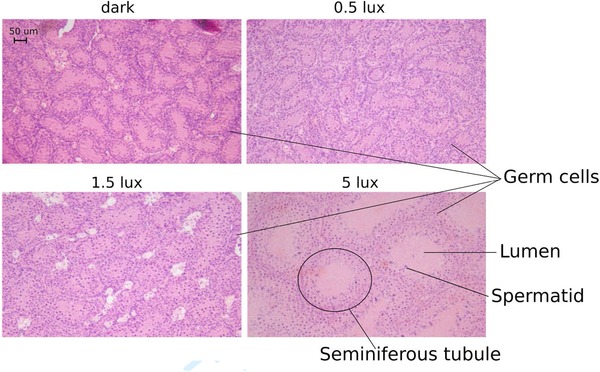
Testis histology. Photomicrographs of representative testes sections for each treatment. A marked increase in the size of the seminiferous tubules (diameter and total area inside the tubule, also called lumen) can be seen in the 5 lux example. The small dots visible in all treatments are germ cells. Complete spermatogenesis can be seen in the 5 lux group by the presence of all stages of germ cell development including elongated spermatids (dark dots) [Color figure can be viewed at http://wileyonlinelibrary.com]

All our models showed a significant increase in testis volume and tubule diameter with increasing light intensity (*P* < 0.001 in both cases, Supporting Information Table S2). For testis volume, this relationship was quadratic, while for tubule diameter this was linear. Post hoc comparisons of estimated means and confidence intervals indicated that there was a slight, nonsignificant increase in testis volume between the dark group and the 0.5 lux group, and a much larger, highly significant increase between 0.5 and 1.5 lux and between 1.5 and 5 lux (Figure 2a, Supporting Information Table S2). Indeed, the testes of the 5 lux birds were six times larger than those of the birds in the 1.5 lux group (Figure 2a and Supporting Information Table S2). Tubule diameter was found to be significantly different between all treatment groups, with a particularly strong increase between 1.5 and 5 lux, although with a smaller effect size compared to the testis volume (Figures 2b and 3, Supporting Information Table S2). Spermatogenesis was detected in all 5 lux testes, but not in the testes of any other treatment group (Figure 3). Age was not a significant predictor of either testis volume (*P* = 0.79) or tubule diameter (0.94).

### 3.2 Gene expression

The analysis of functionally grouped transcripts revealed progressive activation on different stages of gonadal growth with increasing light intensity. Transcript levels of all functional groups analyzed were significantly increased by light exposure (*P* < 0.001 in all cases, Table 2). However, for the germ cell development group, we found significant differences between all levels of the variable treatment, whereas for all other functional groups we found significantly increased transcript levels only at 5 lux (Table 2). In addition, for the germ cell development and the corticoid receptor groups, transcript levels were significantly higher when birds were sacrificed during the day compared to when they were euthanized during the night (Table 2). However, for the corticoid receptors, the effect of time of sacrifice depended on the treatment level (treatment × time interaction, *P* < 0.001): daytime and night‐time transcript levels were similar in the dark control group and in the 0.5 lux group, while at 1.5 and 5 lux, daytime levels were significantly higher than night‐time ones (Table 2).

**Table 2:** Summary of model outputs for gene expression data with transcripts grouped by functional traits. All models were linear mixed models with Gaussian error structure with treatment, time, age, mass and the interaction treatment × time as explanatory variables, and individual ID nested into block (position of a cage within the wall of an experimental room) as random variable, to correct for multiple transcript measurements per individual. All transcript levels were standardized (*z*‐scores) to enable using them as grouped response variable. Post hoc tests were done by comparing the confidence intervals (CI) of the estimates (marginal means). Two levels were considered to be significantly different if the mean estimate for one level was not included in the CI of the other level, and such differences are indicated in the column “Significance”. Reference level for treatment is the dark group, for time it is daytime. Sample sizes: dark: day = 6, night = 7; 0.5 lux: day = 2, night = 5; 1.5 lux: day = 2, night = 5; 5 lux: day = 2, night = 5

Main model						Post‐hoc test					
**Germ cells development (stra8 and spo11)**						**Germ cells development (stra8 and spo11)**					
	Estimate	*SEM*	*df*	*t*	*P*‐value	Treatment	Estimate	*SEM*	Lower CI	Upper CI	Significance
Intercept	−0.37	0.13	30	−2.86	0.008	dark	−0.57	0.09	−0.76	‐0.38	a
Treatment	0.43	0.04	30	11.40	<0.001	0.5	−0.35	0.08	−0.52	−0.18	b
Time	−0.40	0.15	30	−2.66	0.012	1.5	0.08	0.08	−0.07	0.24	c
						5	1.60	0.16	1.28	1.92	d
**Sertoli cells activity (fshr, wt1, sox9, cldn11)**						**Sertoli cells activity (fshr, wt1, sox9, cldn11)**					
Intercept	−0.29	0.10	130	−2.89	0.004	dark	−0.29	0.10	−0.50	−0.09	a
Treatment	0.20	0.04	130	4.83	<0.001	0.5	−0.19	0.09	−0.37	−0.01	a
						1.5	0.01	0.08	−0.15	0.18	b
						5	0.72	0.17	0.37	1.06	c
**Leydig cells activity (lhr and hsd3b1)**						**Leydig cells activity (lhr and hsd3b1)**					
Intercept	−0.93	0.20	30	−4.73	<0.001	dark	−0.35	0.12	−0.61	−0.10	a
Treatment	0.29	0.05	30	5.56	<0.001	0.5	−0.21	0.11	−0.44	0.02	a
Age	0.25	0.07	30	3.45	0.002	1.5	0.08	0.10	−0.13	0.28	b
						5	1.08	0.21	0.65	1.51	c

						Corticoid receptors (gr and mr)						
Corticoid receptors (gr and mr)						Treatment	Time	Estimate	*SEM*	lower CI	upper CI	Significance
Intercept	−0.44	0.16	62	−2.80	0.007	dark	day	−0.44	0.16	−0.76	−0.12	A
Treatment	0.59	0.07	62	8.06	<0.001	0.5	day	−0.14	0.14	−0.43	0.14	A
Time	−0.09	0.20	62	−0.45	0.654	1.5	day	0.45	0.13	0.17	0.72	B
Treatment × Time	−0.35	0.09	62	−3.90	<0.001	5	day	2.52	0.31	1.88	3.15	C
						dark	night	−0.53	0.13	−0.79	−0.27	a
						0.5	night	−0.41	0.11	−0.64	−0.18	a
						1.5	night	−0.17	0.10	−.37	0.04	d
						5	night	0.69	0.20	0.28	1.09	e

**Figure 4:**
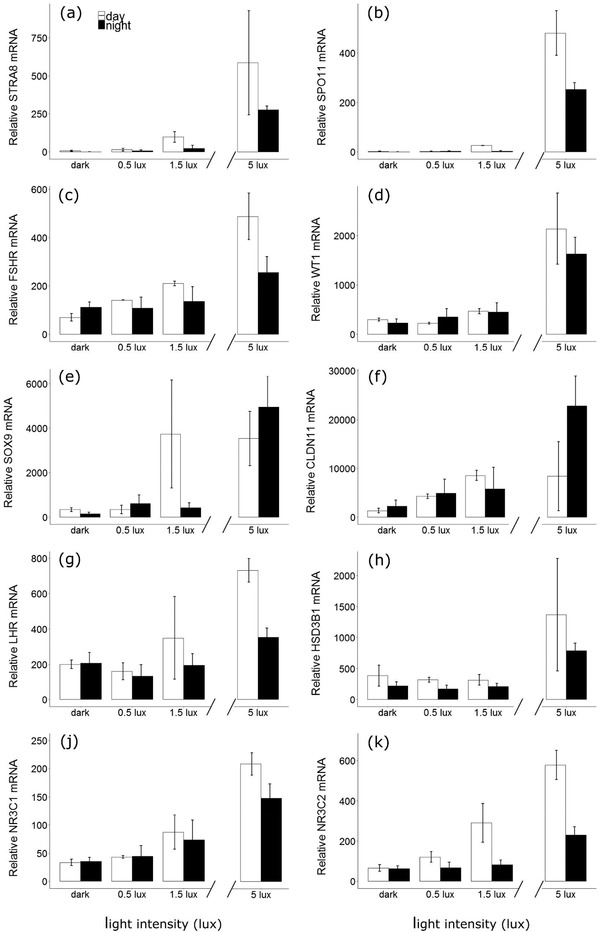
Testicular transcript levels in birds exposed to different levels of light based on real‐time PCR (mean ± *SEM*). Treatment was a continuous variable in all models. Open and closed bars represent birds culled at noon or midnight respectively. Sample sizes: dark: day = 6, night = 7; 0.5 lux: day = 2, night = 5; 1.5 lux: day = 2, night = 5; 5 lux: day = 2, night = 5

The individual analyses of transcript levels of all genes revealed similar patterns to the functional groups analysis. Transcript levels were significantly increased by light exposure (*P* < 0.001 in all cases, Figure 4 and Supporting Information Table S3). For *spo11*, we detected significant changes over all light levels and both at day and at night, while this was not true for *stra8*, the other marker of germ cell development, for which we only found significant changes between 0.5–1.5 lux and 1.5–5 lux.

The general pattern for the other genes indicated that mRNA transcript levels were low and not different between the dark group and the 0.5 lux group. Then, transcript levels increased slightly but significantly between the 0.5 and the 1.5 lux group (except for *hsd3b1*), and finally peaked at 5 lux, with a minimum increase of 20% compared to the 1.5 lux treatment for all genes (Figure 4 and Supporting Information Table S3). The time at which the gonads were harvested had little effect on these transcript levels. The variable time was only found to be significant for *stra8*, *spo11*, and *gr*, which showed reduced transcript levels at night compared to daytime (Supporting Information Table S3), similarly to what we found in the functional group analysis. In addition, for five out of the 10 transcripts measured (*gr*, *lhr*, *fshr*, *hsd3b1*, and *sox9*), there was a positive, significant relationship between age of a bird and transcript levels (Supporting Information Table S3), with older birds showing higher gene expression.

**Figure 5:**
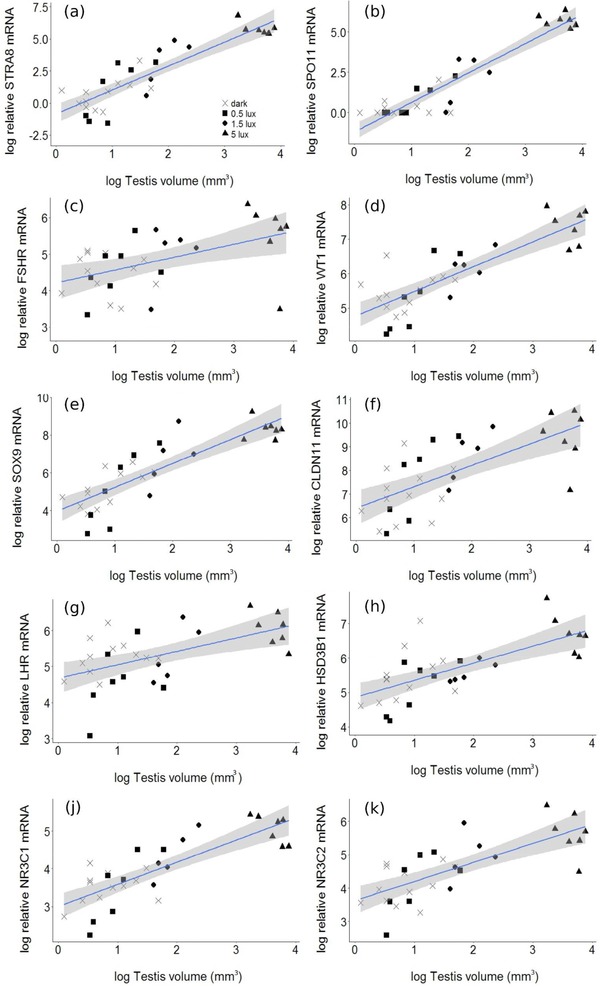
Relationship between testicular volume and relative mRNA transcript levels. Each point in the figures represents one individual bird. Lines and shaded areas represent mean predicted values ± confidence intervals obtained from linear mixed models. Sample sizes were: dark = 13 (crosses), 0.5 lux = 7 (triangles), 1.5 lux = 7 (squares), 5 lux = 7 (circles) [Color figure can be viewed at http://wileyonlinelibrary.com]

In our separate analysis of testis size, we found highly significant, positive relationships between testis volume and transcript levels for all genes analyzed (Supporting Information Table S4 and Figure 5). However, the slope of these relationships was not different between treatments.

## 4 DISCUSSION

In this study, we show that all investigated intensities of ALAN affect the reproductive system of male great tits. Our lowest treatment level, an intensity of 0.5 lux, is comparable to measurements obtained from individual wild birds living in light‐polluted areas (de Jong, Ouyang, van Grunsven, Visser, & Spoelstra, 2016b; Dominoni et al., 2013a). It thus represents realistic levels that birds may encounter in the wild. In particular, we found evidence that different stages of gonadal growth are activated at different levels of light at night. Indeed, mRNA transcript levels of genes linked to the development of germ cells (*stra8* and *spo11*) were already increased under 0.5 lux compared to the dark control group. Germ cell development depends on Sertoli cells activity, but we detected a significant change in the markers of Sertoli cells only under light levels higher than the 0.5 lux group. The explanation for this apparent contradiction might be partly biological and partly methodological. When Sertoli cells are first activated, their response is to induce the proliferation of germ cells. Such proliferation rapidly leads to a much higher number of germ cells, implying a very strong response of germ cell markers compared to those of Sertoli cells. Our method for measuring activation was probably more sensitive to the cumulative transcripts of an increasing numbers of germ cells, as compared to the more gradual activation of Sertoli cells.

**Figure 6:**
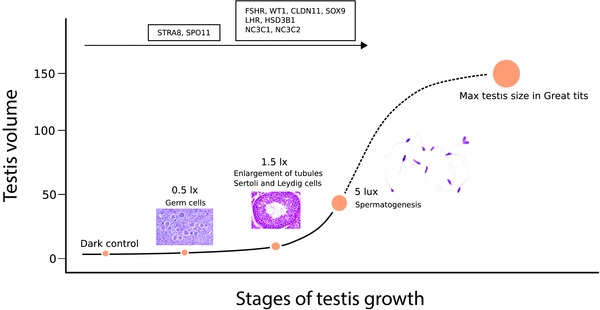
Stages of testis growth under the investigated light intensities, superimposed on a curve of full testicular development. Different processes appear to be activated at different stages of growth due to exposure to ALAN. Germ cell proliferation starts partly already under 0.5 lux, although little changes in testis volume are observed. Under 1.5 lux most of the genes analyzed showed increased transcript levels, and testis tubules started to enlarge, indicating that steroid synthesis and spermatogenesis had started. Full spermatogenesis was only achieved under 5 lux, in parallel with a large increase in testis size. Note that at the time of culling, birds in the 5 lux group had only one‐third of the maximal testis size known from great tits, suggesting that our birds had not yet completed gonadal growth [Color figure can be viewed at http://wileyonlinelibrary.com]

mRNA transcript levels associated with markers of Leydig cell activity were found to be increased only under light levels equal to or higher than 1.5 lux. Indeed, we also found that testes of birds under 1.5 lux were larger than those of birds under 0.5 lux or under dark nights, confirming that a second stage of gonadal development that includes increase in size and the initiation of sperm and steroid production was initiated. However, we only found full spermatogenesis in the histological analyses of birds under 5 lux, which indicates that only birds in this treatment group had functional testes and were thus potentially ready to breed (Figure 6). These birds also showed a dramatic increase in testes size, as they had 10 to 30 times larger testes than birds kept at lower light intensities, as well as greatly increased mRNA levels of all transcripts analyzed (Figure 6). Therefore, while exposure to ALAN as low as 0.5 lux during our experiment already induced a photoperiodic response in great tits, only levels equal to 5 lux led to full spermatogenesis. However, despite the fact that birds in the 5 lux group had far larger testes than birds in any other treatment group and also showed full spermatogenesis, at the time of sampling, the birds were still far from having reached the average and maximum testis volume of this species (130 and 150 mm^3^, Figure 6 and Schaper et al., 2012a, b).

A gradual increase of effects with increasing levels of light at night for gonadal development parallels our findings for daily rhythms (de Jong et al., 2016a). When the same individual birds were exposed to similar levels of ALAN during the preceding experiment, activity patterns and nocturnal melatonin concentration were also affected in a dose‐dependent manner. The earlier study also reported slight but significant effects at lower light intensities, and more marked effects under 5 lux (de Jong et al., 2016a). In particular, the strong advancement of the morning onset of activity found under 5 lux in the previous study (figure 2a in de Jong et al., 2016a) is comparable to the strong increase in testes size and gene expression that we show here for the 5 lux group. Over the course of these two studies, the birds were exposed twice to their respective ALAN conditions, interrupted for 3 weeks by exposure to dark nights. It is therefore possible that the reproductive activation we measured had been primed before the start of our experiment (Sockman, Williams, Dawson, & Ball, 2004; te Marvelde et al., 2012). However, given the intermittent pausing of the ALAN treatments, we consider it likely that the main effects detected in our present study had developed during our 3‐week experiment in February. We also need to stress that our experimental findings are specific to the photosensitive phase of the annual cycle. It is currently unclear what effects temporary exposure to ALAN will have at other phases of the annual cycle. In a previous experiment, in which we exposed blackbirds to ALAN for an entire annual cycle, we found that ALAN advanced reproductive development, as in this study. Under continued exposure to ALAN, blackbirds regressed testes at similar times as dark‐night controls. However, under ALAN, birds did not recover photosensitivity and remained locked in the photorefractory phase until at least the following summer. Associated physiological processes, i.e., postbreeding moult, were also impaired by persistent exposure to ALAN (Dominoni, Quetting, & Partecke, 2013b).

A limitation of our sampling design is that we have obtained only a single‐point measurement during the gonadal growth of great tits. It is therefore impossible to obtain information about the growth curve of each individual's reproductive system, which would have been helpful to establish more precisely the shape of the dose‐dependent response of the reproductive system to ALAN. On the other hand, our terminal experiment allowed us to obtain tissue samples for histological and molecular analyses. Future studies should attempt to use more treatment groups and repeatedly measure gonadal size in the same individuals, to understand the exact shape of the dose‐response of reproductive growth to ALAN. In our experiment we found that testis volume was correlated at the individual level to mRNA transcript levels, suggesting that it is possible to infer from consecutive measures of testicular size of living birds the underlying molecular processes (Partecke et al., 2005).

Interestingly, the increase in testis volume due to ALAN was also strongly linked to increased mRNA levels of two adrenal steroid receptors, *GR* and *MR*. In birds, glucocorticoids have been suggested to mediate timing of reproduction via the regulation of steroid hormone synthesis (Crespi et al., 2013; Deviche et al., 2010; Goutte et al., 2010a, b; Kirby et al., 2009; McGuire et al., 2013; Schoech, Mumme, & Wingfield, 1997, 2009). Indeed, recent studies have shown that acute stressors and the resulting heightened circulating corticosterone levels are able to lower testosterone and estradiol release in Rufous‐winged sparrows (*Peucaea carpalis*) and European starlings (*Sturnus vulgaris*) (Deviche et al., 2010; McGuire et al., 2013). However, these manipulations did not result in changes in the gonadal expression of GnRH in the starling study (McGuire et al., 2013). Moreover, recent work in captive dark‐eyed juncos (*Junco hyemalis thurberi*) has also questioned the direct effect of glucocorticoids on gonadal processes (Fudickar et al., 2017). While we did not measure corticosterone, the ratio of *MR/GR*, which is often used to test for altered stability of the stress axis (Marasco, Herzyk, Robinson, & Spencer, 2016), did not vary between the four treatments (Supporting Information Figure S2a), and individual *gr* and *mr* transcript levels were highly correlated (Supporting Information Figure S2b). These data seem to indicate that the birds in our experiment were not stressed, and thus provide little support for a negative influence of ALAN on the HPA axis. Rather, they suggest that higher levels of adrenal steroid receptors are likely a by‐product of major metabolic changes accompanying, or even preparing for, important life‐history transitions, as shown for avian migration (Piersma, Reneerkens, & Ramenofsky, 2000) and for reproduction (this study).

One additional factor that affected mRNA levels was the age of birds. An advancement of reproductive activation with increasing age is common in birds and has also been linked to age‐related changes in photic sensitivity (Sockman et al., 2004). However, in our birds only mRNA levels of few genes were related to age, and both testis volume and tubule diameter were not. Thus, whether the effect of ALAN on reproductive development of wild birds might be age‐specific, and particularly affects older birds, remains to be established.

From previous work on avian species, exposure to ALAN is known to advance both the development of the reproductive system and egg‐laying (Dominoni, 2015). Indeed, in blackbirds, experimental exposure to 0.3 lux of ALAN for 8 weeks in captivity caused birds to develop fully functional testes (Dominoni et al., 2013a). Although exposure was longer and methods were not directly comparable to those in our study, it is possible that blackbirds are particularly sensitive to ALAN, matching reports of species‐specific differences (Da Silva, Samplonius, Schlicht, Valcu, & Kempenaers, 2014; Kempenaers et al., 2010). To explore possible differences in sensitivity to ALAN, future work could empirically compare the physiological responses of different avian species, both closely and distantly related, to increasing levels of light at night.

In an ecological context, it is still unclear whether earlier development of the reproductive system due to ALAN would lead to earlier breeding. In wild great tits, the advancement of lay date due to ALAN is limited to only a few days. Moreover, such an effect seems to be modulated by temperature, being stronger in cold and late springs compared to warmer ones (de Jong et al., 2015). This refines reported relationships between warmer temperatures in urban areas (the “heat island effect”) and avian reproductive timing (Deviche & Davies, 2014). In any case, in our experiment, temperature was kept equal across all treatments and rooms, thus the observed variation in reproductive timing is mostly attributable to the light treatments. However, although ALAN can be perceived as a long photoperiod (Dominoni & Partecke, 2015) and lead to earlier gonadal development, individual variation in reproductive physiology is not necessarily related to individual variation in egg‐laying dates. Indeed, recent experimental work in great tits has shown that although long photoperiods lead to increased secretion of reproductive hormones and larger gonads, this was unrelated to subsequent egg‐laying dates (Salis et al. in review; Schaper et al., 2012a; te Marvelde et al., 2012). To our knowledge, no study has attempted to simultaneously measure the timing of both gonadal growth and egg‐laying in response to ALAN, and this remains a considerable research gap. Further comparisons can be made also to studies performed in other taxa. Indeed, in a similar experiment with a freshwater fish species (European perch), Bruening and collaborators (Bruening et al., 2016) also showed intensity‐dependent effects of ALAN on the expression of gonadotropins (LH and FSH), although with two key differences compared to our study. First, they only found an effect in females but not males, and second, they found that gonadotropin expression decreased, rather than increased, with higher light intensity at night. This latter difference can be explained by the different reproductive strategies of the two species. Our work in great tits was conducted in late winter when great tits are photosensitive and may be expected to show reproductive activation under ALAN, as the presence of light at night may be interpreted as a stimulating photoperiod in long‐day avian breeders (Dominoni & Partecke, 2015; Dominoni et al., 2013a). Conversely, perch are short‐day breeders, which require shortening photoperiods to initiate reproductive activity (Migaud, Wang, Gardeur, & Fontaine, 2006, 2010). ALAN‐induced perceived long photoperiods are known to suppress gonadotropins in this species and other short‐day breeders (Bruening, Hölker, Franke, Preuer, & Kloas, 2015; Robert et al., 2015). Overall, the studies suggest that the photoperiodic response of birds and fish to ALAN is dose‐dependent. The presence of ALAN is globally increasing in both spatial extent and radiance, despite the recent switch to LED technology in developed countries (Falchi et al., 2016; Gaston, Duffy, & Bennie, 2015a; Kyba et al., 2017). This suggests that exposure to ALAN of wild animals is likely also increasing, and thus there is impellent need to understand what the ecological consequences of ALAN will be in the coming years (Davies & Smyth, 2018). Our study adds to the increasing evidence that the exposure to artificial light can affect the reproductive system of animals, and in particular of birds. Our study was aimed at understanding in detail the physiological pathways involved in the stimulation of the reproductive system by ALAN, as well as potential thresholds above which the reproductive response is triggered. It remains to be established whether not only seasonal processes, but also diel ones, can be profoundly affected at the tissue level by low levels of light at night (de Jong et al., 2016a). In our experiment, we also collected other tissue samples, such as brain, liver, and spleen, which are key regulators of daily rhythms in sleep, metabolism, and immunity, but these data will be published separately (Dominoni et al in prep). From this present work we conclude that even light levels as low as 0.5 lux can produce early gonadal activation, although only higher light intensities of at least 5 lux were able to strongly increase testes size and lead to full spermatogenesis within our experimental period. Wild birds are likely to be exposed to such levels in light polluted areas (de Jong et al., 2016b; Dominoni et al., 2013a, 2014). For some species exposure as in our captive experiment might not last for the entire night. However, others, in particular animals living in open areas, will be less able to “escape” light pollution, as shown in wallabies (Robert et al., 2015). Thus, we argue that ALAN should be limited to minimal levels wherever possible to avoid chronically high exposure for wildlife. However, to build a stronger case for the negative effects of light pollution on wildlife, and thus to support the implementation of novel policies aimed at limiting ALAN, future work should not only examine the behavioral and physiological effects of light pollution, but also clarify whether these come with health and fitness consequences (de Jong et al., 2015; Dominoni, Borniger, & Nelson, 2016; Kempenaers et al., 2010; McLay, Green, & Jones, 2017; Ouyang et al., 2017; Swaddle et al., 2015).

## Supporting information

Supporting Information
