# Supplementary material for: Dose‐response effects of light at night on the reproductive physiology of great tits (Parus major): Integrating morphological analyses with candidate gene expression
Source: J Exp Zool A Ecol Integr Physiol. 2018 Jul 29;329(8-9):473–87. doi: 10.1002/jez.2214 (PMC6220976; doi:10.1002/jez.2214)
Supplement: Supplementary file 1 — Supporting Information [file JEZ-329-473-s001.pdf]

Supplementary online material for:

Dose-response effects of light at night on the reproductive physiology of great tits (*Parus major*): integrating morphological analyses with candidate gene expression

Davide M. Dominoni, Maaike de Jong, Michelle Bellingham, Peter O'Shaughnessy, Kees van Oers, Jane Robinson, Bethany Smith, Marcel E. Visser, Barbara Helm

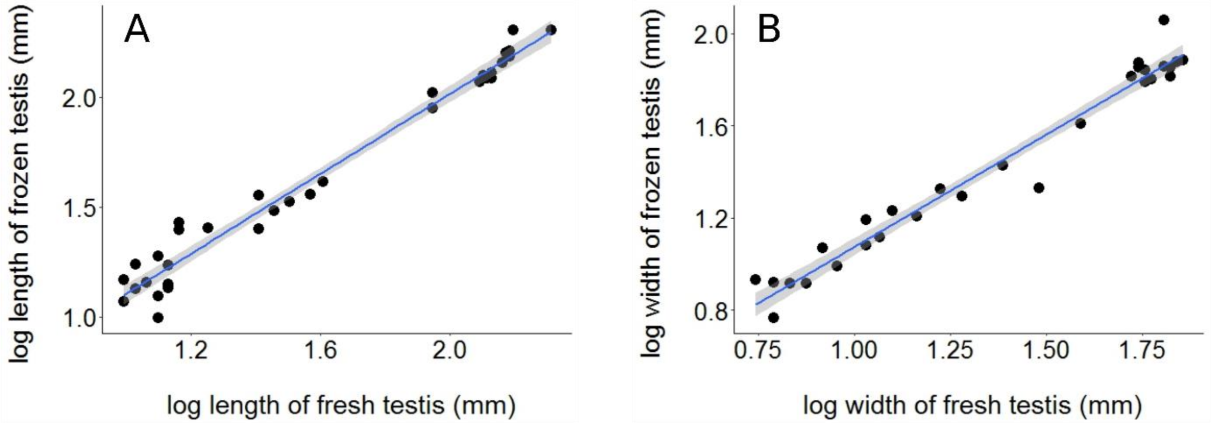

Figure S1. Correlation between fresh and frozen measurements of testis length (A) and testis width (B) obtained from great tits from a separate experiment (data courtesy of Irene Verhagen)

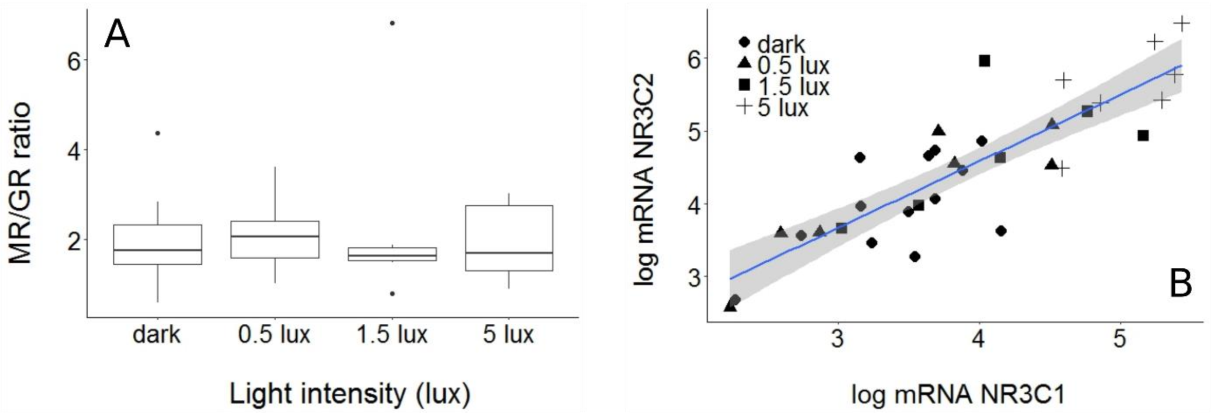

Figure S2. Panel A: Variation in the ratio between MR (*NR3C2*) and GR (*NR3C1*) mRNA transcript levels in the testes between the four treatments was not significant (p = 0.753). Panel B: GR and MR transcript levels were correlated within individuals (p <0.001).

Table S1: List of primers used in the experiment.

Gene name	Gene acronym	Forward primer	Reverse primer
Hydroxy-delta-5-steroid dehydrogenase, 3 beta- and steroid delta-isomerase 1	HSD3B1	aagaggaggcgctggctgaga	gatgacgagggagacgccctg
Luteinizing hormone receptor	LHR	gagtgtgaaagcaccatgaggaaacc	agccggccatttcgctgtc
Follicle stimulating hormone receptor	FSHR	gcaaacttcacctatcctagccattgc	tctgtagtcttctgccaggcttctcc
Claudin-11	CLDN11	tgcggctacaccatcaccacc	gcgatcatcagcgctcgacat
Wilms tumor protein	WT1	cacagcttgaatgcatgacatggaatc	cacgtcttgtatgcctctaaaaactccg
SRY (Sex Determining Region Y)-Box 9	SOX9	gctccagcaagaacaaaccgcac	tctcgctctcgttcagcagcct
SPO11, initiator of meiotic double stranded breaks	SPO11	gtgaattgtacctgcagtgcaacagc	ctttattgaagaagtcatcatccaggagtctc
STRA 8, stimulated by retinoic acid gene 8	STRA8	tatccccagatctagtggaatttgaacg	tccctgctctcttcagaaagctcttg
Nuclear receptor subfamily 3, group C, member 1	NR3C1 (GR)	attggctccgctgggaacg	aggcctcgtcagagcacacca
Nuclear receptor subfamily 3, group C, member 2	NR3C2 (MR)	tgtgtctgtcatcgtttgccttgag	cggacgaactgcaggctgatct

Table S2. Summary of model outputs for the morphological and histological data collected on testes. All models were linear mixed models with Gaussian error structure with treatment, age and mass as explanatory variables, and block (position of a cage within the wall of an experimental room) as random effect nested into room. Model selection was conducted by backward, step-wise removal of non-significant variables. Post-hoc tests were done by comparing the confidence intervals (CI) of the estimates for each level of the continuous variable treatment or the factor time. Two levels were considered to be significantly different if the mean estimate for one level was not included in the CI of the other level, and such differences are indicated by different letters in the column “sig”. Reference level for treatment is the dark group, for time it is daytime.

Main model

Tubule diameter

Parameter	Estimate	SEM	df	t	p
Intercept	49.00	4.76	11	10.29	<0.001
Treatment	26.96	1.77	24	15.20	<0.001

Testis volume

Parameter	Estimate	SEM	df	t	p
Intercept	2.55	1.29	18	1.98	0.063
Treatment	-0.16	1.99	25	-0.08	0.938
Treatment^2	1.45	0.38	25	3.80	<0.001

Post-hoc test

Tubule diameter

Treatment	Estimate	SEM	lower CI	upper CI	sig
dark	49.00	4.78	38.56	59.45	a
0.5	62.48	4.35	52.98	71.99	b
1.5	89.45	3.97	80.76	98.13	c
5	183.82	7.39	167.67	199.97	d

Testis volume

Treatment	Estimate	SEM	lower CI	upper CI	sig
dark	2.55	1.29	-0.17	5.27	a
0.5	2.84	1.04	0.64	5.03	a
1.5	5.58	1.70	2.01	9.16	b
5	38.05	1.81	34.24	41.87	c

Table S3. Summary of model outputs for individual gene expression data. All models were linear mixed models with Gaussian error structure with treatment, time, age, mass and the interaction treatment*time as explanatory variables, and block (position of a cage within the wall of an experimental room) as random variable. Post-hoc tests were done as indicated in Table S2.

Main model / **Post-hoc test**

STRA8

Parameter	Estimate	SEM	df	t	p
Intercept	1.66	0.45	18	3.66	0.002
Time	-1.89	0.47	28	-3.99	<0.001
Treatment	1.11	0.11	25	9.88	<0.001

STRA8

Treatment	Time	Estimate	SEM	lower CI	upper CI	sig
dark	day	1.66	0.46	0.69	2.62	a
0.5	day	2.22	0.45	1.28	3.16	a
1.5	day	3.33	0.44	2.40	4.26	b
5	day	7.23	0.62	5.93	8.54	c
dark	night	0.23	0.41	-1.10	0.64	d
0.5	night	0.33	0.39	-0.50	1.15	d
1.5	night	1.44	0.37	0.67	2.22	a
5	night	5.34	0.53	4.24	6.45	e

SPO11

Parameter	Estimate	SEM	df	t	p
Intercept	0.61	0.25	24	2.40	0.024
Time	-0.69	0.29	30	-2.35	0.026
Treatment	1.11	0.07	25	15.23	<0.001

SPO11

Treatment	Time	Estimate	SEM	lower CI	upper CI	sig
dark	day	0.61	0.26	0.07	1.15	a
0.5	day	1.16	0.25	0.64	1.68	b
1.5	day	2.27	0.25	1.76	2.78	c
5	day	6.14	0.37	5.37	6.92	d
dark	night	0.08	0.22	-0.54	0.38	e
0.5	night	0.47	0.21	0.05	0.90	a
1.5	night	1.58	0.19	1.19	1.97	c
5	night	5.46	0.31	4.82	6.09	d

FSHR

Parameter	Estimate	SEM	df	t	p
Intercept	3.93	0.26	23	14.97	<0.001
Treatment	0.24	0.06	25	3.93	0.001
Age	0.22	0.09	26	2.50	0.019

FSHR

Treatment	Estimate	SEM	lower CI	upper CI	sig
dark	4.43	0.19	4.01	4.85	a
0.5	4.55	0.17	4.16	4.94	a
1.5	4.79	0.16	4.42	5.15	b
5	5.62	0.27	5.01	6.23	c

WT1

Parameter	Estimate	SEM	df	t	p
Intercept	5.33	0.18	9	29.10	<0.001
Treatment	0.41	0.05	25	7.67	<0.001

WT1

Treatment	Estimate	SEM	lower CI	upper CI	sig
dark	5.33	0.18	4.91	5.74	a
0.5	5.53	0.17	5.13	5.93	ab
1.5	5.94	0.17	5.56	6.32	b
5	7.36	0.25	6.79	7.94	c

SOX9

Parameter	Estimate	SEM	df	t	p
Intercept	4.04	0.40	25	10.12	<0.001
Treatment	0.69	0.10	25	6.96	<0.001
Age	0.45	0.14	27	3.21	0.003

SOX9

Treatment	Estimate	SEM	lower CI	upper CI	sig
dark	5.08	0.27	4.49	5.67	a
0.5	5.43	0.25	4.89	5.96	ab
1.5	6.11	0.23	5.61	6.61	b
5	8.52	0.42	7.60	9.44	c

CLDN11

Parameter	Estimate	SEM	df	t	p
Intercept	7.13	0.28	31	25.81	<0.001
Treatment	0.48	0.12	31	4.21	<0.001

CLDN11

Treatment	Estimate	SEM	lower CI	upper CI	sig
dark	7.13	0.28	6.53	7.73	a
0.5	7.37	0.25	6.84	7.91	a
1.5	7.86	0.22	7.38	8.34	b
5	9.55	0.47	8.54	10.56	c

LHR

Parameter	Estimate	SEM	df	t	p
Intercept	4.31	0.20	30	21.24	<0.001
Treatment	0.22	0.05	30	4.15	<0.001
Age	0.29	0.07	30	3.96	<0.001

LHR

Treatment	Estimate	SEM	lower CI	upper CI	sig
dark	5.00	0.13	4.72	5.28	a
0.5	5.11	0.12	4.86	5.36	ab
1.5	5.33	0.10	5.10	5.55	b
5	6.10	0.22	5.63	6.57	c

HSD3B1

Parameter	Estimate	SEM	df	t	p
Intercept	4.63	0.21	30	21.93	<0.001
Treatment	0.29	0.06	30	5.34	<0.001
Age	0.26	0.08	30	3.44	0.002

HSD3B1

Treatment	Estimate	SEM	lower CI	upper CI	sig
dark	5.25	0.13	4.96	5.53	a
0.5	5.49	0.12	5.13	5.65	a
1.5	5.69	0.11	5.45	5.92	b
5	6.72	0.23	6.23	7.21	c

GR

Parameter	Estimate	SEM	df	t	p
Intercept	3.05	0.21	25	14.62	<0.001
Treatment	0.34	0.05	25	6.58	<0.001
Age	0.18	0.07	27	2.52	0.018

GR

Treatment	Estimate	SEM	lower CI	upper CI	sig
dark	3.47	0.14	3.16	3.78	a
0.5	3.64	0.13	3.35	3.93	a
1.5	3.98	0.12	3.71	4.24	b
5	5.15	0.22	4.67	5.63	c

MR

Parameter	Estimate	SEM	df	t	p
Intercept	4.42	0.22	20	20.17	<0.001
Time	-0.66	0.23	29	-2.80	0.009
Treatment	0.35	0.06	25	6.22	<0.001

MR

Treatment	Time	Estimate	SEM	lower CI	upper CI	sig
dark	day	4.42	0.22	3.96	4.89	a
0.5	day	4.60	0.22	4.14	5.05	ab
1.5	day	4.95	0.21	4.50	5.39	b
5	day	6.17	0.31	5.53	6.81	c
dark	night	3.77	0.20	3.35	4.18	d
0.5	night	3.94	0.19	3.55	4.33	d
1.5	night	4.29	0.18	3.92	4.66	ad
5	night	5.51	0.26	4.97	6.05	e

Table S4. Summary of models testing for the relationship between testis volume and gene transcript levels (both log-transformed). All models were linear mixed models with Gaussian error structure with testis volume, treatment, age, mass and the interaction treatment*testis volume as explanatory variables, and block (position of a cage within the wall of an experimental room) as random effect nested into room.

**STRA8**					
	**Estimate**	**SEM**	**df**	**t**	**p**
(Intercept)	-0.84	0.37	27	-2.28	0.031
log(Testis.volume)	1.87	0.18	24	10.44	< 0.001

**SPO11**					
	**Estimate**	**SEM**	**df**	**t**	**p**
(Intercept)	-0.57	0.33	28	-1.71	0.098
log(testis volume)	0.98	0.34	28	2.89	0.007

**FSHR**					
	**Estimate**	**SEM**	**df**	**t**	**p**
(Intercept)	-1.20	0.25	29	-4.71	< 0.001
log(testis volume)	1.83	0.12	29	14.73	< 0.001

**WT1**					
	**Estimate**	**SEM**	**df**	**t**	**p**
(Intercept)	4.76	0.19	17	24.56	< 0.001
log(testis volume)	0.72	0.08	24	9.47	< 0.001

**SOX9**					
	**Estimate**	**SEM**	**df**	**t**	**p**
(Intercept)	3.96	0.31	29	12.74	< 0.001
log(testis volume)	1.27	0.15	29	8.36	< 0.001

**CLDN11**					
	**Estimate**	**SEM**	**df**	**t**	**p**
(Intercept)	6.40	0.36	29	17.61	< 0.001
log(testis volume)	0.90	0.18	29	5.10	< 0.001

**LHR**					
	**Estimate**	**SEM**	**df**	**t**	**p**
(Intercept)	4.68	0.21	29	22.57	< 0.001
log(testis volume)	0.37	0.10	29	3.68	0.001

**HSD3B1**					
	**Estimate**	**SEM**	**df**	**t**	**p**
(Intercept)	4.86	0.19	25	25.11	< 0.001
log(testis volume)	0.50	0.09	24	5.27	< 0.001

GR					
	**Estimate**	**SEM**	**df**	**t**	**p**
(Intercept)	3.00	0.16	25	18.59	< 0.001
log(testis volume)	0.59	0.08	24	7.69	< 0.001

MR					
	**Estimate**	**SEM**	**df**	**t**	**p**
(Intercept)	3.63	0.20	25	17.94	< 0.001
log(testis volume)	0.57	0.10	24	5.88	< 0.001
